# Special Characteristics and Synchronizations of Multi Hybrid-Order Chaotic Systems

**DOI:** 10.3390/e22060664

**Published:** 2020-06-16

**Authors:** Jiaxun Liu, Zuoxun Wang, Fangfang Zhang, Yankai Yin, Fengying Ma

**Affiliations:** 1 School of Electrical Engineering and Automation, Qilu University of Technology (Shandong Academy of Sciences), Jinan 250353, China; 1043117185@stu.qlu.edu.cn (J.L.); 1043117180@stu.qlu.edu.cn (Z.W.); 1043117198@stu.qlu.edu.cn (Y.Y.); mfy@qlu.edu.cn (F.M.); 2 Shandong Computer Science Center (National Supercomputer Center in Jinan), Shandong Artificial Intelligence Institute, Qilu University of Technology (Shandong Academy of Sciences), Jinan 250101, China; 3 College of Control Science and Engineering, Shandong University, Jinan 250061, China

**Keywords:** hybrid order chaotic systems, fractional order, hybrid degree, combination synchronization

## Abstract

Based on advantages of integer and fractional chaotic systems, hybrid chaotic systems and their definitions and some fundamental concepts are proposed, such as hybrid degree (HD), the lowest order (LO) and the total dimension order (TDO). The preliminary properties of hybrid Lorenz systems and hybrid forms of some classic chaotic systems are studied. Then, the relations between HD, LO and TDO with different parameters is investigated in chaotic systems. To be specific, HD is associated with fractional order. It is a directional method to search LO and TDO in chaotic systems. Finally, based on the incommensurate fractional stability theory, we accomplish combination synchronization for three different hybrid order chaotic systems. The simulation results verify the effectiveness of the synchronization controller.

## 1. Introduction

Since Lorenz proposed the first concrete chaotic system in the modeling of weather forecasts in 1963 [1], chaos, as a significant branch of nonlinearity communities, has been a very hot topic until now. It is known that the evolutions of chaotic systems sensitively rely on the special initial conditions and parameters, as two identical chaotic systems start from slightly different initial conditions or parameters can separate exponentially with time. It is the interesting features and its potential applications in image encryptions, pseudo random number generators and secure communications [2,3,4,5,6] that enable numerous researchers to devote in finding the new chaotic models, for instance, enhanced integer chaotic maps, hidden attractors [7,8,9] and integer conservative chaotic systems. Chen chaotic system is applied to produce random sequences. Using these sequences, some arrays were created for image permutation and key stream production in Reference [10]. A generalized multilevel-hybrid chaotic oscillator (GM-HCO) was created by combining a multilevel discrete function generated from user data with a continuous function having a damping factor greater than ln(2) to achieve variable rates and adaptive carrier frequencies [11]. By means of different hybrid chaotic sequences and systems, an image segmentation encryption was presented in Reference [12]. However, as the anti-cryptography technology develops, a host of chaotic communications by means of integer chaotic system were not safe, especially in the narrow keyspace and simple chaotic dynamics.

The fractional-order differential equations can date back to Leibniz’s note in 1695 at the earliest. Due to the weakness in engineering background, fractional-order differential equations always existed in pure mathematic fields. The importance of fractional-order differential equations were valued by many scholars untill the last decades, since the fractional-order differential equations have more accurate descriptions in some real systems and it can give a clear insight into the physical processes underlying a long range memory, such as the fractional-order differential equations in the model of human immunodeficiency virus [13], fractional market dynamics [14] and viscoelastically damped structures [15]. In 1995, Hartley combined the fractional-order differential equations and Chua system and got the fractional Chua chaotic system [16]. Subsequently, Grigorenko [17] introduced the fractional Lorenz system and found that the dimension order of fractional system is lower than integer systems in the domain of chaotic behaviors. Fractional Chen [18] and Lü [19] were also presented successively. Luo extended the complex Lorenz system into the fractional complex Lorenz system and analyzed its complicated dynamic characteristics [20]. Based on the Chen system, the generation of a class of hyperchaotic systems are studied using both integer order and fractional-order differential equation systems in Reference [21], which provide a new method to create chaotic attractors by means of switching control.

The mentioned articles made some great innovations in generalization of chaotic systems and secure communications based on fractional chaos [2]. Compared with integer chaotic system, the fractional chaotic system with fractional order can regard the fractional order as another type of the secret key in the process of image, video encryptions and secure communications [22,23,24]. Therefore, fractional order chaotic systems have advantages over integer chaotic system in complicated keyspace and dynamic behaviors. However, it is the existence of fractional order that make some extra difficulties in the hardware implementation [25,26,27,28], such as FPGA. Especially, the fractional-order differential is more complicated than integer-order differential in circuit design. Therefore, an interesting question appears. Can the chaotic system own both merits of integer and fractional chaotic system? Specifically, the hybrid order chaotic system is the balance of fractional and integer order chaotic system. It means part of state variables are integer order differential, and the other state variables are fractional-order differential. Not only does it own the complex keyspace and dynamic behaviors in secure communications, but also it can be easily implemented in realization.

Besides, in the process of exploring chaos, the parameters of chaotic systems are vital for discovering chaotic domain. To be specific, some intrinsic relations between state variables and chaotic parameters illustrate the existence of chaotic attractors, such as the positive Lyapunov indexs, the Feigenbaum contant and the bifurcation diagrams. However, when nonlinear systems exist chaotic dynamics, the potential relations among different parameters are seldom studied, which is a new field to deeply understand chaos.

In addition, the phenomenon of synchronization universally exists in nature, such as frogs croak together and fireflies glow together. Chaos synchronization, as a part of chaos control, has arisen numerous attention in the last 30 years. Some significant synchronization forms were successively proposed, such as complete synchronization [29], projective synchronization [30], function projective synchronization [31] and complex modification projective synchronization [32]. Mohadeszadeh achieved hybrid chaos synchronization between two identical complex and two identical real fractional-order chaotic systems employing fractional-order sliding mode control approach [33]. In light of the interaction among several financial factors, Reference [34] use integer order and fractional-order differential equation systems to model a financial system and based on proposed fractional financial chaotic systems, the fractional chaos synchronization was achieved by relevant controller. Note that in the process of fractional chaos synchronization, all the fractional order is identical in the numerical simulations. However, in practical chaotic systems, the fractional orders are not always equal to each other. Thus, it is meaningful to focus on incommensurate fractional chaotic multi-system synchronization.

Motivated by the above discussions, some innovations are obtained in this paper. (1) Firstly, to combine the advantages of integer and fractional chaotic systems, the definitions of hybrid order chaotic systems are proposed. Hybrid order chaotic system is as easy to implement as the integer order chaotic system. It also has the intricate chaotic dynamic behaviors and complicated parameters, which is good to secure communications in sense of chaotic systems. (2) Then, by means of numerous analyses in hybrid order systems, such as hybrid order Lorenz systems, hybrid order Chen system, hybrid order Lü system and hybrid order complex Lorenz, a universal relation between hybrid degree (HD) and fractional order is presented, and bifurcation diagrams reinforce the belief. (3) Lastly, hybrid order chaotic systems belong to inhomogeneous fractional order system in essence. We design the controller for combination synchronization. Relevant simulations verify the effectiveness of proposed controllers.

The rest structure of this paper is organized as follows. In Section 2, we present some basic mathematical background and definitions of hybrid order chaotic systems. The property analyses and common relations in hybrid order chaotic systems are studied in Section 3. We focus on the combination synchronization on different hybrid order chaotic systems in Section 4. Finally, the conclusion is drew in Section 5.

## 2. Mathematical Background

Fractional-order differential equations mainly have three definitions, such as the Riemann– Liouville definition, the Grunwald–Letnikov definition and the Caputo definition. As traditional initial conditions and expressions of constants are included in Caputo definition, we use it in this paper.

**Definition** **1** ([35])**.** *The Caputo fractional derivative is shown as follows,*

(1) $$D^{q}f\left(t\right)=\frac{1}{\Gamma (p-q)}\int_{q}^{t}{\left(t-\tau \right)}^{p-1-q}f^{p}\left(\tau \right)d\tau ,$$

*where p=[q]+1,[q] is the integer part of q, Γ(∗) is the gamma function and $D^{q}$ is called q-order differential operator.*

Then, we give the definition and some concepts of hybrid order chaotic system.

**Definition** **2.** *For a m-dimension chaotic system*

(2) $$D^{q_{\gamma }}x_{\gamma }=Ax+f\left(x\right),$$

*where $q_{\gamma }$ is the order of state variables $x_{\gamma }$, γ=1,2,…,m, x is state vairables matrix, **A** is linear matrix of chaotic system and f(x) is the nonliner part.*

*If it exists at least one γ that $q_{\gamma }=1$ and at least one γ that $q_{\gamma }$ is fractional order, then the m-dimension chaotic system is called hybrid order chaotic system. The number of equations whose order is $q_{\gamma }=1$ is Hybrid Degree (HD). The lowest order (**LO**) indicates the lowest fractional order in all fractional state variables, and the total dimension order (**TDO**) means the sum of order for m equations.*

**Definition** **3.** *For a m-dimension integer chaotic system.*

(3) $$\left\{\begin{array}{cc} {\dot{x}}_{1} & =g_{1}\left(x_{1},x_{2},\ldots ,x_{m}\right),\\ {\dot{x}}_{2} & =g_{2}\left(x_{1},x_{2},\ldots ,x_{m}\right),\\ \vdots \\ {\dot{x}}_{m} & =g_{m}\left(x_{1},x_{2},\ldots ,x_{m}\right), \end{array}\right.$$

*where $g_{1}\left(*\right),g_{2}\left(*\right),\ldots ,g_{m}\left(*\right)$ are corresponding continuous functions and $R^{m}\to R^{m}$. The value of hybrid forms of Equation (3) is Hybrid Number $\mathrm{HN}=C_{m}^{1}+C_{m}^{2}+\ldots +C_{m}^{m-1}$, where $C_{c}^{d}=\frac{c!}{(c-d)d!}$ and c,d are two constants (c≥d).*

**Lemma** **1.** *Consider a n-dimension autonomous fractional system*

(4) $$D^{q}x\left(t\right)=Wx\left(t\right),x\left(0\right)=x_{0},$$

*where $x\in R^{n},0<q\leq 1,W\in R^{n\times n}$. $D^{q}=\left[D^{q_{1}},D^{q_{2}},\ldots ,D^{q_{n}}\right]$ demonstrates the fractional derivatives order q. And let $q=\frac{a}{b}$, where a,b∈N,gcd(a,b)=1, M is the lowest common multiples of all denominators b.*

***(1)** [36] If $q_{1}=q_{2}=\cdots =q_{n}$, system Equation (4) is a commensurate fractional order system. Equation (4) is asymptotically stable if and only if*

(5) $$\arg\left(\lambda \right)\geq \frac{q\pi }{2},$$

*where λ are all eigenvalues of Jacobian matrix **J** of Equation (4).*

***(2)** [37] If q are not identically equal to each other, system Equation (4) is a incommensurate fractional order system. Equation (4) is asymptotically stable if and only if*

(6) $$\arg\left(\lambda \right)\geq \frac{\pi }{2M},$$

*where all λ meet following equation*

(7) $$\det(\mathrm{diag}\left(\left[\lambda^{q_{1}M},\lambda^{q_{2}M},\ldots ,\lambda^{q_{n}M}\right]\right)-J)=0.$$

## 3. Characteristics Analysis of Hybrid Order Chaotic Systems

### 3.1. Hybrid Order Lorenz Systems

In this part, we present the hybrid order Lorenz systems and study its chaotic characteristics by means of bifurcation diagrams and other numerical analysis and computer simulation methods. An interesting finding that the relation between fractional order *q* and HD is preliminarily obtained.

Based on the classic integer Lorenz chaotic system, we choose hybrid order Lorenz systems with HD = 1, which is shown as follows,

(8) $$\left\{\begin{array}{cc} D^{q_{1}}x & =w_{1}\left(y-x\right),\\ D^{q_{2}}y & =w_{3}x-xz-y,\\ \dot{z} & =xy-w_{2}z, \end{array}\right.$$

(9) $$\left\{\begin{array}{cc} D^{q_{1}}x & =w_{1}\left(y-x\right),\\ \dot{y} & =w_{3}x-xz-y,\\ D^{q_{3}}z & =xy-w_{2}z, \end{array}\right.$$

(10) $$\left\{\begin{array}{cc} \dot{x} & =w_{1}\left(y-x\right),\\ D^{q_{2}}y & =w_{3}x-xz-y,\\ D^{q_{3}}z & =xy-w_{2}z. \end{array}\right.$$

where $w_{1}=10$, $w_{2}=8/3,w_{3}=28$.

When HD=2, then

(11) $$\left\{\begin{array}{cc} D^{q_{1}}x & =w_{1}\left(y-x\right),\\ \dot{y} & =w_{3}x-xz-y,\\ \dot{z} & =xy-w_{2}z, \end{array}\right.$$

(12) $$\left\{\begin{array}{cc} \dot{x} & =w_{1}\left(y-x\right),\\ D^{q_{2}}y & =w_{3}x-xz-y,\\ \dot{z} & =xy-w_{2}z, \end{array}\right.$$

(13) $$\left\{\begin{array}{cc} \dot{x} & =w_{1}\left(y-x\right),\\ \dot{y} & =w_{3}x-xz-y,\\ D^{q_{3}}z & =xy-w_{2}z. \end{array}\right.$$

Therefore, the possible forms $\mathrm{HN}=C_{3}^{1}+C_{3}^{2}=6$. It is in accord with Definition 3.

#### 3.1.1. Dissipativeness and the Existence of Equilibriums

Owning to the consistency of right side of hybrid Lorenz systems, we consider their dissipativeness as follows,

(14) $$\begin{array}{cc} ▽V & =-w_{1}-1-w_{2}\\ \; & =-10-1-8/3<0. \end{array}$$

It demonstrates that hybrid Lorenz systems are dissipative systems and is similar with integer Lorenz system.

#### 3.1.2. Equilibriums and Stability

Note that the calculation of equilibriums depends on the right side of chaotic systems, six forms of hybrid Lorenz systems have the same equilibriums. Then we set

(15) $$\left\{\begin{array}{c} w_{1}\left(y-x\right)=0,\\ w_{3}x-xz-y=0,\\ xy-w_{2}z=0, \end{array}\right.$$

where $w_{1}=10,w_{2}=\frac{8}{3},w_{3}=28$. There are three equilibriums (0,0,0), $\left(6\sqrt{2},6\sqrt{2},27\right)$ and $\left(-6\sqrt{2},-6\sqrt{2},27\right)$. In order to predict stability of equilibriums, we give the Jacobian matrix of Equation (15) as follows,

$$\left[\begin{array}{ccc} -10 & 10 & 0\\ 28-z & -1 & -x\\ y & x & -\frac{8}{3} \end{array}\right]$$

Substitute equilibriums into Jacobian matrix respectively, then eigenvalues are −22.8277,11.8277,−2.6667 with equilibrium (0,0,0), and eigenvalues are −13.8546,0.0940+10.1945i,0.0940−10.1945i when equilibriums are $\left(\pm 6\sqrt{2},\pm 6\sqrt{2},27\right)$. Thus, these three equilibriums are unstable.

#### 3.1.3. Bifurcations of Hybrid Order Lorenz Systems

Diagrams of bifurcation is significant label in chaotic behavior, which can present the relation between state variables and parameters. In this section, we aim at studying the potential law in different parameters by means of bifurcation diagrams. 

(1) **HD** = 1 

**Figure 1:**
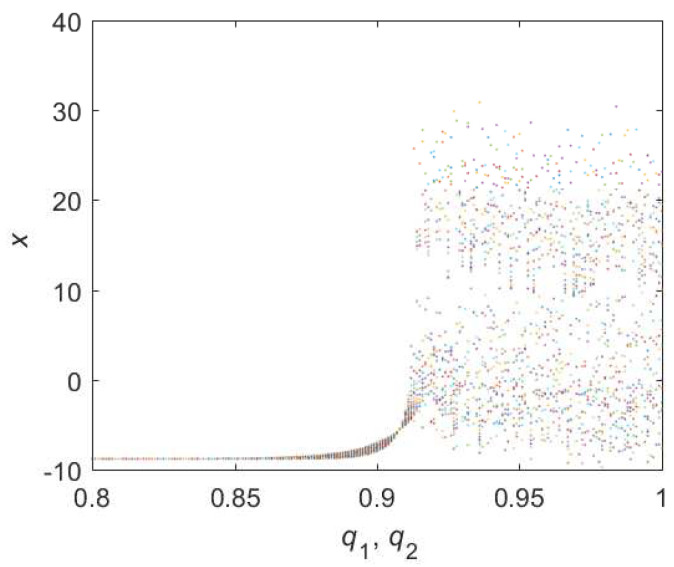
Bifurcation diagram of hybrid order Equation (8) with the initial condition is (0.1,0.5,0.5), step-size is 0.0195 and the number of iterations is 1000.

**Figure 2:**
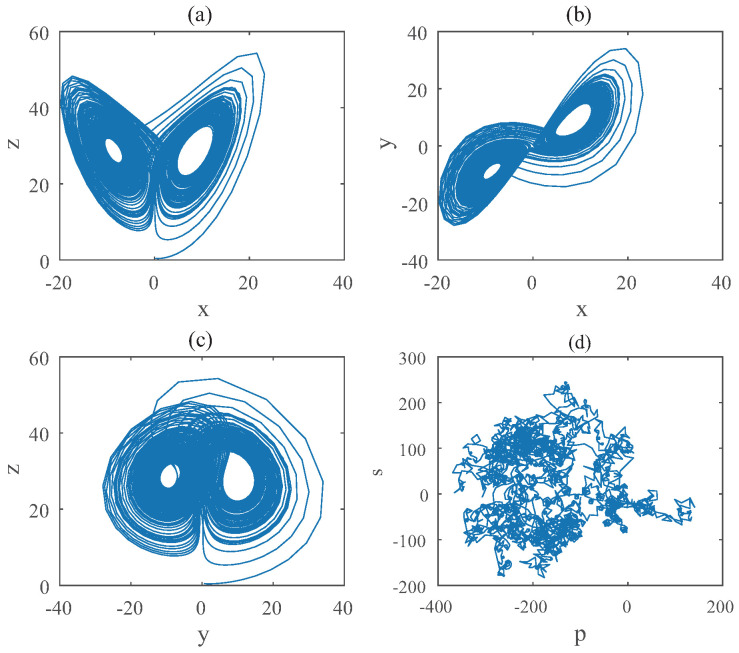
States diagram of hybrid order Equation (8), where the initial condition is (0.1,0.5,0.5), $q_{1}=q_{2}=0.91,q_{3}=1$, step-size is 0.0195, the number of iterations is 3000 and (**a**) is x−z, (**b**) is x−y, (**c**) is y−z and (**d**) is 0-1 test diagram.

We set that all the initial condition as (0.1,0.5,0.5) and step-size as 0.0195. For Equation (8), the bifurcation diagram is shown in Figure 1. From the diagram one can know that the chaotic domain appears in $q_{1}=q_{2}=0.91$. And the 0-1 test diagram and phase pictures of $q_{1}=q_{2}=0.91$ are shown in Figure 2.

The brownian motion and chaotic attractors demonstrate the existence of chaotic dynamics. Therefore, the lowest order (LO) is q=0.91 and total dimension order TDO is 1+0.91+0.91=2.82, approximately.

**Figure 3:**
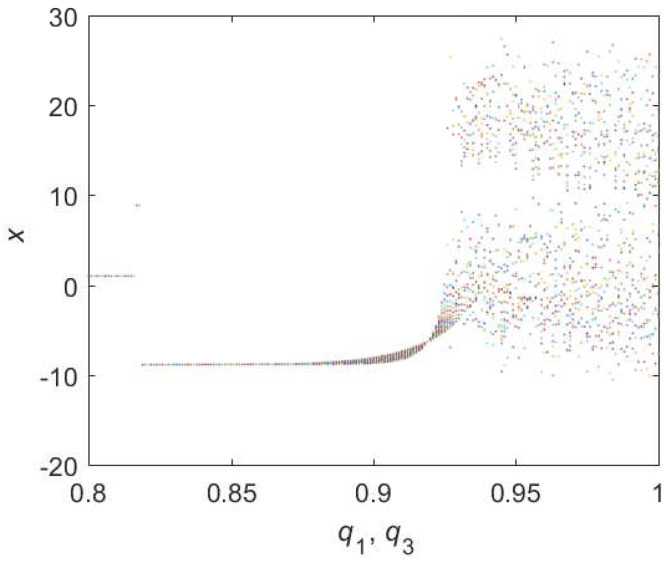
Bifurcation diagram of hybrid order Equation (9) with the initial condition is (0.1,0.5,0.5), step-size is 0.0195 and the number of iterations is 1000.

**Figure 4:**
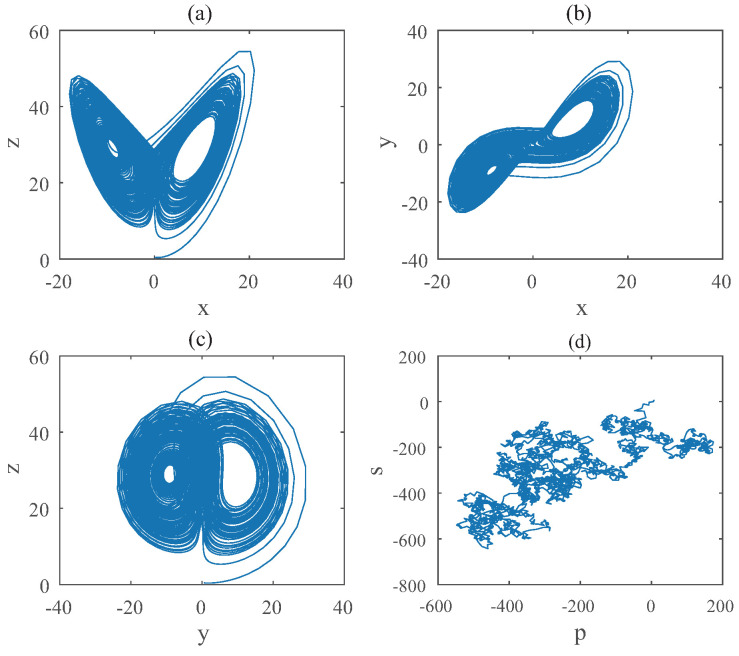
States diagram of hybrid order Equation (9), where the initial condition is (0.1,0.5,0.5), $q_{1}=q_{3}=0.93,q_{2}=1$, step-size is 0.0195, the number of iterations is 3000 and (**a**) is x−z, (**b**) is x−y, (**c**) is y−z and (**d**) is 0-1 test diagram.

For Equation (9), the bifurcation diagram is shown in Figure 3. From the diagram we can get that the chaotic domain appears in $q_{1}=q_{3}=0.93$. And the 0-1 test diagram and phase pictures of $q_{1}=q_{2}=0.93,q_{3}=1$ are shown in Figure 4, which indicate the existence of chaotic area. In this case, the LO is q=0.93 and TDO is 1+0.93+0.93=2.86, approximately.

**Figure 5:**
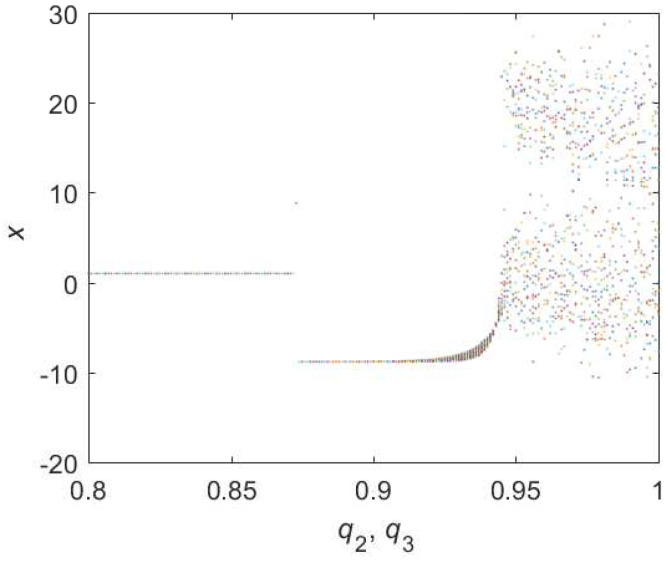
Bifurcation diagram of hybrid order Equation (10) with the initial condition is (0.1,0.5,0.5), step-size is 0.0195 and the number of iterations is 1000.

**Figure 6:**
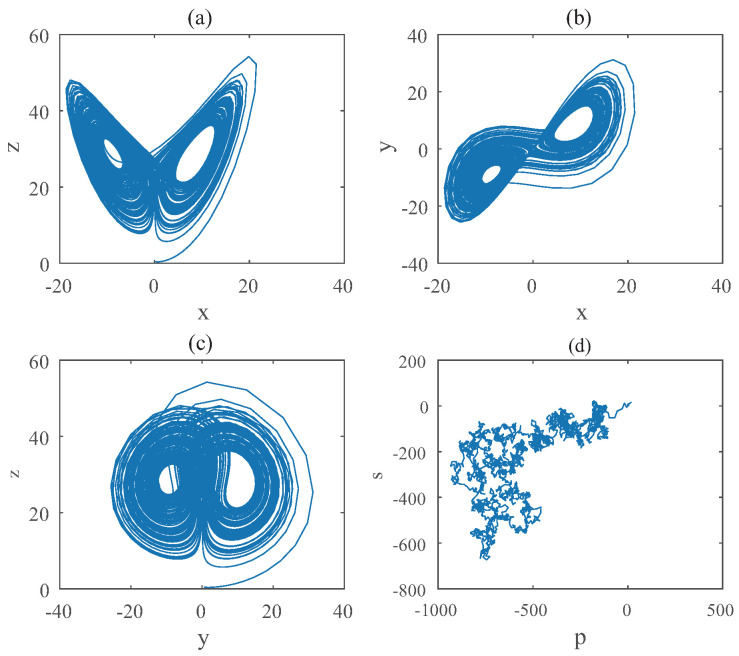
States diagram of hybrid order Equation (10), where the initial condition is (0.1,0.5,0.5), $q_{1}=1,q_{2}=q_{3}=0.95$, step-size is 0.0195, the number of iterations is 3000 and (**a**) is x−z, (**b**) is x−y, (**c**) is y−z and (**d**) is 0-1 test diagram.

When it comes to Equation (10), the bifurcation diagram is shown in Figure 5. Chaotic domain appears in $q_{2}=q_{3}=0.95$. And the 0-1 test diagram and phase pictures of $q_{2}=q_{3}=0.95$ are shown in Figure 6. Chaotic dynamics encounter in the Equation (10) with $q_{2}=q_{3}=0.95$. Thus, the LO is q=0.95 and TDO is 1+0.95+0.95=2.9 in this case, approximately.

Based on the above results of three types of chaotic systems, it is not difficult to find that the lowest LO is q=0.91 and the lowest TDO is 1+0.91+0.91=2.82 in hybrid order Lorenz system with HD=1.

(2) HD=2

**Figure 7:**
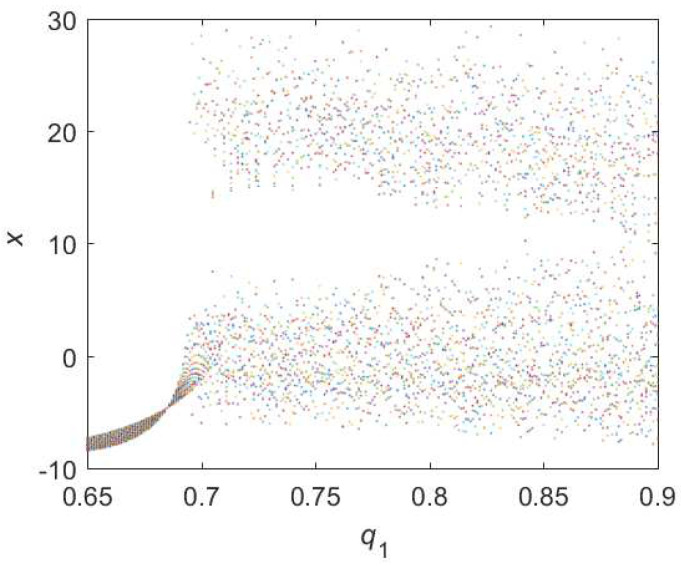
Bifurcation diagram of hybrid order Equation (11) with the initial condition is (0.1,0.5,0.5), step-size is 0.0195 and the number of iterations is 1000.

**Figure 8:**
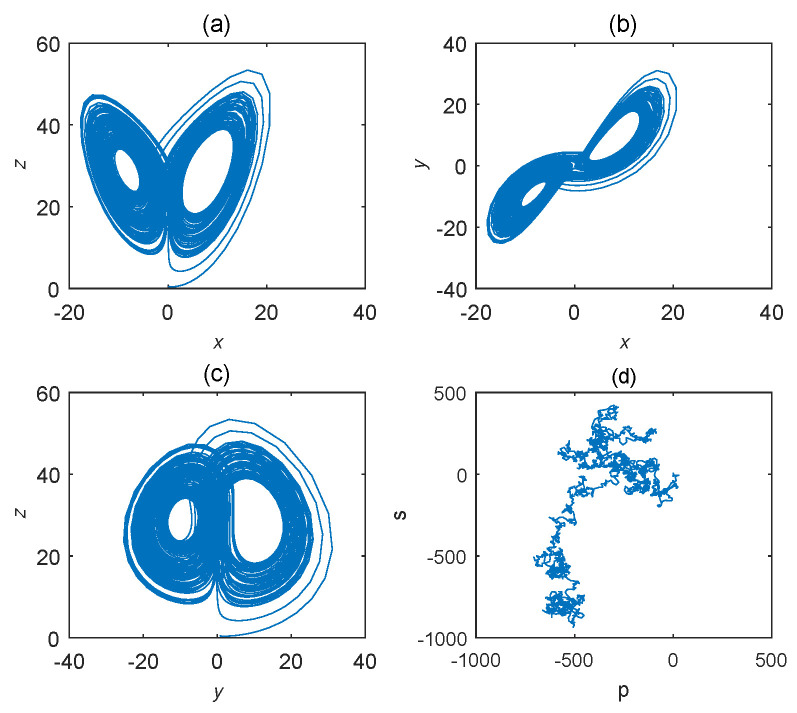
States diagram of hybrid order Equation (11), where the initial condition is (0.1,0.5,0.5), $q_{1}=0.7,q_{2}=q_{3}=1$, step-size is 0.0195, the number of iterations is 3000 and (**a**) is x−z, (**b**) is x−y, (**c**) is y−z and (**d**) is 0-1 test diagram.

As for Equation (11), the bifurcation diagram is shown in Figure 7. From the diagram, chaotic domain appears in $q_{1}=0.7$. And the 0-1 test diagram and phase pictures of $q_{1}=0.7$ are shown in Figure 8. The brownian motion and chaotic attractors demonstrate the existence of chaotic dynamics. Therefore, the LO is q=0.7 and TDO is 1+1+0.7=2.7, approximately.

**Figure 9:**
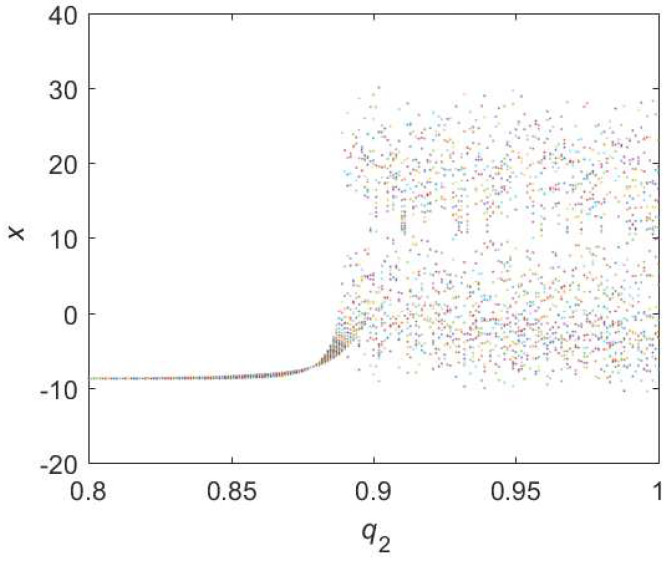
Bifurcation diagram of hybrid order Equation (12) with the initial condition is (0.1,0.5,0.5), step-size is 0.0195 and the number of iterations is 1000.

**Figure 10:**
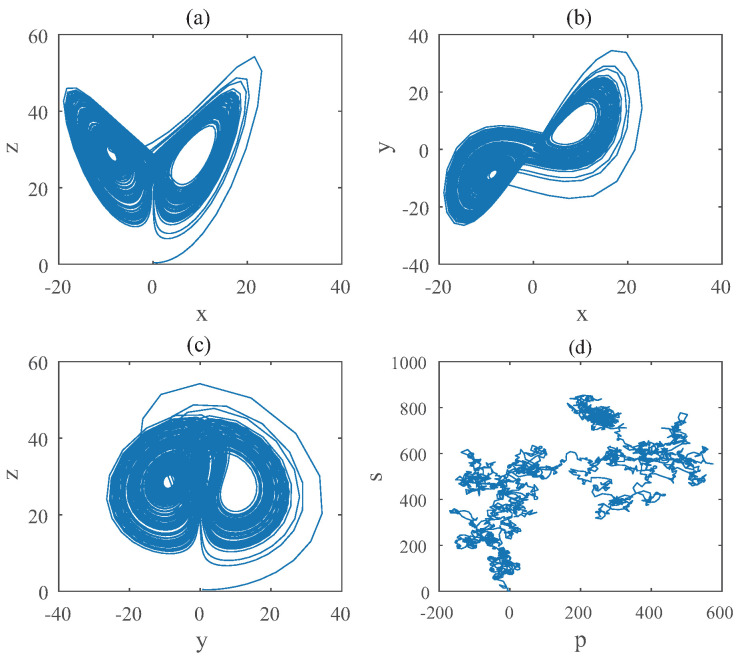
States diagram of hybrid order Equation (12), where the initial condition is (0.1,0.5,0.5), $q_{2}=0.7,q_{1}=q_{3}=1$, step-size is 0.0195, the number of iterations is 3000 and (**a**) is x−z, (**b**) is x−y, (**c**) is y−z and (**d**) is 0-1 test diagram.

For Equation (12), the diagram of bifurcation is shown in Figure 9. Chaotic domain appears in $q_{2}=0.89$. And the 0-1 test diagram and phase picture of $q_{2}=0.89$ are shown in Figure 10, which indicate the existence of chaotic area. In a nutshell, the **LO** is q=0.89 and TDO is 1+0.89+1=2.89 in this case, approximately.

**Figure 11:**
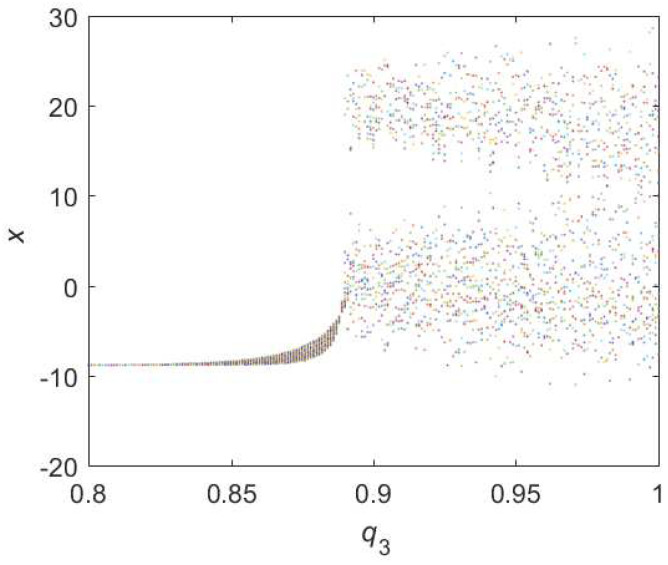
Bifurcation diagram of hybrid order Equation (13) with the initial condition is (0.1,0.5,0.5), step-size is 0.0195 and the number of iterations is 1000.

**Figure 12:**
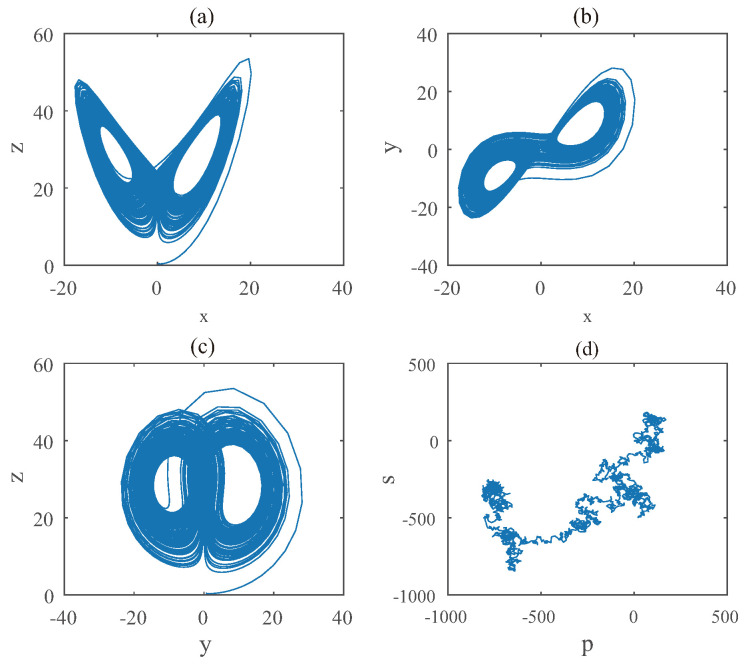
States diagram of hybrid order Equation (13), where the initial condition is (0.1,0.5,0.5), $q_{1}=q_{2}=1,q_{3}=0.89$, step-size is 0.0195, the number of iterations is 3000 and (**a**) is x−z, (**b**) is x−y, (**c**) is y−z and (**d**) is 0-1 test diagram.

When it comes to Equation (13), the diagram of bifurcation is shown in Figure 11. One can see that the chaotic domain appears when $q_{3}=0.89$. And the 0-1 test diagram and phase pictures of $q_{3}=0.89$ are shown in Figure 12. The brownian motion and chaotic attractors demonstrate the existence of chaotic dynamics. Therefore, the LO is q=0.89 and TDO is ∑=1+1+0.89=2.89 in this case, approximately.

Based on bifurcation analyses of Equations (11)–(13), the lowest LO is q=0.7 and the lowest TDO is 1+1+0.7=2.7.

(3) HD=0

**Figure 13:**
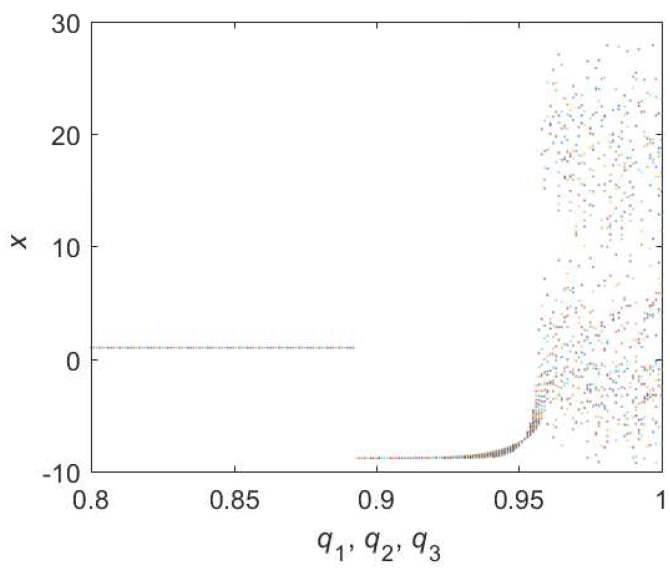
Bifurcation diagram of hybrid order Equation (16) with the initial condition is (0.1,0.5,0.5), step-size is 0.0195 and the number of iterations is 1000.

The basic form of complete fractional Lorenz system is shown in Equation (16). To compare with hybrid Lorenz systems, bifurcation diagram of complete fractional Lorenz system ($q_{1}=q_{2}=q_{3}$) is also obtained in Figure 13. If it exists $0<q_{1},q_{2},q_{3}<1$, the chaotic domain is appeared in $q_{1}=q_{2}=q_{3}=0.97$. It is in accordance with Reference [17]. Namely, the lowest TDO is 0.97+0.97+0.97=2.91 and the lowest LO is q=0.97.

(16) $$\left\{\begin{array}{cc} D^{q_{1}}x & =w_{1}\left(y-x\right),\\ D^{q_{2}}y & =w_{3}x-xz-y,\\ D^{q_{3}}z & =xy-w_{2}z, \end{array}\right.$$

#### 3.1.4. Relations of Different Chaotic Parameters in Hybrid Lorenz Systems

In order to get a clear comparison result of HD, LO and TDO, each of indexes of hybrid order Lorenz systems and complete fractional order Lorenz system are shown in Table 1.

Inspired by the above simulation results, one can recognize some interesting findings. When HD=0, the lowest TDO=2.91 and the lowest LO=0.97; When HD=1, the lowest TDO is 2.82 and the lowest LO is 0.91; When HD=2, the lowest TDO is 2.7 and the lowest LO is 0.7. It can be seen that when 0≤HD<m, as the increase of HD, the lowest LO and the lowest TDO reduce gradually. Specifically, in hybrid Lorenz systems, there always exists inverse ratio relations between HD with LO and TDO. As we increase the value of HD, we always find the lower TDO than complete fractional chaotic system in hybrid Lorenz systems. Detailedly, complete fractional Lorenz system’s TDO is 2.91, while the lowest TDO is 2.82 for hybrid Lorenz systems with HD=1 and the lowest TDO is 2.7 for HD=2. These two cases have smaller TDO than complete fractional Lorenz system’s HD = 0. Therefore, we can find much lower order hybrid chaotic system. It demonstrates that some intrinsic relations between integer order and fractional order chaotic systems are discovered. This finding shock us greatly and another question appears. Generally, do all hybrid chaotic systems meet this rule?

### 3.2. Other Classic Hybrid Order Chaotic ystems

In this part, numerous hybrid forms of some classic chaotic systems are obtained to testify the relation between HD, LO and TDO. In order to get an explicit result, we do not present detailed chaotic dynamic analyses of every hybrid order systems and give the values of HD, LO and TDO in some tables.

#### 3.2.1. Hybrid Order Chen Systems

Hybrid order Chen systems are shown in Table 2. When HD=0, the fractional order is the same and the lowest order is q=0.78, TDO=0.78+0.78+0.78=2.34. With the increase of HD, the lowest LO is 0.68 and the lowest TDO is 2.36. Then, we continue to add the value of HD. Excitedly, when HD=2, the lowest LO is 0.275, and the lowest TDO is 1+1+0.275=2.275.

To be specific,

HD=0, the lowest LO is 0.78, the lowest TDO is 2.34;

HD=1, the lowest LO is 0.68, the lowest TDO is 2.36;

HD=2, the lowest LO is 0.275, the lowest TDO is 2.275.

When we increase the value of HD, the lowest LO gradually decline. Compared with HD=0, we can find a lower TDO=2.275 when HD=2.

#### 3.2.2. Hybrid Order Lü Systems

Hybrid order Lü systems are shown in Table 3, and we have

HD=0, the lowest LO is 0.78, the lowest TDO is 2.34.

HD=1, the lowest LO is 0.653, the lowest TDO is 2.306.

HD=2, the lowest LO is 0.277, the lowest TDO is 2.277.

When we increase the value of HD, the lowest LO and TDO gradually decline. In hybrid order Lü systems, HD has inverse ratio relation with the lowest LO and the lowest TDO. In other words, compared with **HD** = 0, there are two lower TDO in HD=1 and HD=2. It is the same to hybrid order Lorenz systems.

#### 3.2.3. Hybrid Order Complex Lorenz Systems

When it comes to complex fields of chaos, hybrid order complex Lorenz systems are shown in Table 4, and we get

HD=0, the lowest LO is 0.959, the lowest TDO is 2.877.

HD=1, the lowest LO is 0.908, the lowest TDO is 2.816.

HD=2, the lowest LO is 0.531, the lowest TDO is 2.531.

We can see as the value of HD increases, the lowest LO and the lowest TDO gradually decline. It means both the lowest LO and the lowest TDO have inverse ratio relation with HD in hybrid order complex Lorenz systems, which is the same as hybrid Lü systems and hybrid Lorenz systems.

### 3.3. A Relation between Chaotic Parameters for Different Hybrid Order Chaotic Systems

By means of the above bifurcation analyses in different hybrid order chaotic systems, some common relations are found. In hybrid order Lorenz, hybrid order Lü and hybrid order complex Lorenz systems, as increase the value of HD, the lowest LO and the lowest TDO are gradually decreasing. In hybrid Chen systems, the HD has inverse ratio relation with **LO** and we can also find a lower TDO when 0≤HD<m. Therefore, we get

**Conjecture** **1.** *In hybrid order Lorenz, Chen, Lü and complex Lorenz chaotic systems, there exist common relations in hybrid degree (HD), the lowest LO and the lowest TDO. Specifically,*

*(1)*  *HD always has inverse ratio relation with the lowest LO when 0≤HD<m*.
*(2)*  *Compared with complete fractional chaotic systems (HD=0), we can always find lower TDO in hybrid order systems*.

Due to limitation of whole paper, other hybrid order forms of classic chaotic systems, such as Liu, Rossler, Hyperlorenz, Sprott and others, are not detailedly depicted. And same results are obtained in the relations between two parameters of hybrid order systems. By so many simulation experiments, we statistically and preliminarily demonstrate the conjecture. It is advisable for us to increase the **HD** of hybrid forms to seek lower total dimension order and lowest order without complete simulation experiments. It is greatly meaningful to build models in describing some real systems, such as elastic systems, economy systems, model of human immunodeficiency virus and so on.

## 4. Combination Synchronization of Hybrid Order Chaotic Systems

### 4.1. Combination Synchronization

As the special structure features of hybrid order chaotic systems, we can naturally understand that it is associated with incommensurate fractional systems. Some frequently-used theorems, for instance Equation (4) is not adapted to hybrid order chaotic systems. Therefore, it is essential for us to use different theorems to guarantee the achievement of chaos synchronization.

Then, we recall the basic form of combination synchronization [38]. Consider two drive systems and one response system.

The first drive system is

$$x=g_{1}\left(x\right),$$

the second drive system is

$$y=g_{2}\left(y\right),$$

and the response system is

$$z=g_{3}\left(z\right),$$

where $x={\left[x_{1},x_{2},\ldots ,x_{n}\right]}^{T},y={\left[y_{1},y_{2},\ldots ,y_{n}\right]}^{T},z={\left[z_{1},z_{2},\ldots ,z_{n}\right]}^{T}$. All state variables are observable.

They are said to be combination synchronization if it exists three constant matrixes $Q_{1},Q_{2},Q_{3}\in R^{n}$ and $Q_{3}\neq 0$ such that

(17) $$\lim_{t\to +\infty }\left|\right|Q_{3}z-Q_{1}x-Q_{2}y\left|\right|=0,$$

where ||∗|| is the matrix norm.

Case1. When $Q_{3}=1,$$Q_{2}=0,Q_{1}=1$, combination synchronization will be complete synchronization.

Case2. When $Q_{3}=1,$$Q_{2}=0$, combination synchronization will be projective synchronization.

Case3. When $Q_{3}=1,$$Q_{2}=0,Q_{1}=0$, combination synchronization will be chaos control.

We respectively choose one of hybrid Chen and hybrid Lü systems as a group of drive systems, which are shown as follows. The first drive system (hybrid Chen) is

(18) $$\left\{\begin{array}{cc} D^{q_{r}}x_{2} & =35(y_{2}-x_{2}),\\ \dot{y_{2}} & =-7x_{2}-x_{2}z_{2}+28y_{2},\\ \dot{z_{2}} & =x_{2}y_{2}-3z_{2}, \end{array}\right.$$

the second drive system (hybrid Lü) is

(19) $$\left\{\begin{array}{cc} D^{q_{r}}x_{3} & =36(y_{3}-x_{3}),\\ \dot{y_{3}} & =-x_{3}z_{3}+28.7y_{3},\\ \dot{z_{3}} & =x_{3}y_{3}-3z_{3}. \end{array}\right.$$

And the response system (hybrid Lorenz) is

(20) $$\left\{\begin{array}{cc} D^{q_{r}}x_{1} & =10\left(y_{1}-x_{1}\right)+u_{1},\\ \dot{y_{1}} & =28x_{1}-x_{1}z_{1}-y_{1}+u_{2},\\ \dot{z_{1}} & =x_{1}y_{1}-\frac{8}{3}z_{1}+u_{3}, \end{array}\right.$$

where $u_{1},u_{1},u_{3}$ are the corresponding controllers.

Setting $Q_{1}=Q_{2}=Q_{3}=I$ (*I* is an unit matrix), $e_{1}=x_{1}-x_{2}-x_{3},e_{2}=y_{1}-y_{2}-y_{3},e_{3}=z_{1}-z_{2}-z_{3}$, we can get the error system, which is shown as follows,

(21) $$\left\{\begin{array}{cc} D^{q_{r}}e_{1} & =-25y_{2}-26y_{3}+35x_{2}+36x_{3}+10e_{2}+u_{1},\\ \dot{e_{2}} & =35x_{2}+28x_{3}+28e_{1}-x_{2}z_{3}-x_{2}e_{3}-x_{3}z_{2}-\\ \; & \;\;\;\;x_{3}e_{3}-e_{1}z_{2}-e_{1}z_{3}-e_{1}e_{3}-29y_{2}-29.7y_{3}-\\ \; & \;\;\;\;e_{2}+u_{2},\\ \dot{e_{3}} & =x_{2}y_{3}+x_{2}e_{2}+x_{3}y_{2}+x_{3}e_{2}+e_{1}y_{2}+e_{1}y_{3}+\\ \; & \;\;\;\;e_{1}e_{2}+\frac{1}{3}z_{2}+\frac{1}{3}z_{3}-\frac{8}{3}e_{3}+u_{3}. \end{array}\right.$$

Transform Equation (21) to matrix form, then

(22) $$D^{q}e=g\left(x,y,z\right)+u,$$

where $q={\left[q_{r},1,1\right]}^{T},e={\left[e_{1},e_{2},e_{3}\right]}^{T},x={\left[x_{1},x_{2},x_{3}\right]}^{T},y={\left[y_{1},y_{2},y_{3}\right]}^{T},z={\left[z_{1},z_{2},z_{3}\right]}^{T},u={\left[u_{1},u_{2},u_{3}\right]}^{T}$ in this part.

Our purpose is to find an appropriate controller u, which can make

$$\lim_{t\to +\infty }\left||e(t)|\right|=0.$$

Based on active control method, we get the following Theorem.

**Theorem** **1.** *Consider the combination synchronization controller of hybrid chaotic systems*

(23) u=−g(x,y,z)+ke,

*where $\arg\left(\lambda \right)\geq \frac{\pi }{2M}$, all the λ meet following $\det(\mathrm{diag}\left(\left[\lambda^{q_{1}M},\lambda^{q_{2}M},\ldots ,\lambda^{q_{n}M}\right]\right)-J)=0,$ and λ is all eigenvalue of error system with controller, k is the control strength.*

**Proof.**  Substituting controller (23) into error system (22), we get

(24) $$\begin{array}{cc} D^{q}e & =g(x,y,z)-g(x,y,z)+\mathrm{ke}\\ \; & =\mathrm{ke}. \end{array}$$

Since $\det(\mathrm{diag}\left(\left[\lambda_{k}^{q_{1}M},\lambda_{k}^{q_{2}M},\ldots ,\lambda_{k}^{q_{n}M}\right]\right)-J)=0$ and $\arg\left(\lambda_{k}\right)\geq \frac{\pi }{2M}$, it meets Lemma 1. Thus, the error system (22) can be asymptotically stable with the controller (23). □

### 4.2. Simulations

According to the proposed controller in combination synchronization of hybrid chaotic systems, we can give the detailed form of controller, which is shown as follows,

(25) $$\left\{\begin{array}{cc} u_{1} & =-10y_{1}+10x_{1}+35y_{2}-35x_{2}+36y_{3}-36x_{3}-\\ \; & \;\;\;\;k_{1}\left(x_{1}-x_{2}-x_{3}\right),\\ u_{2} & =-28x_{1}+x_{1}z_{1}+y_{1}-7x_{2}-x_{2}z_{2}+28y_{2}-\\ \; & \;\;\;\;x_{3}z_{3}+28.7y_{3}-k_{2}\left(y_{1}-y_{2}-y_{3}\right),\\ u_{3} & =-x_{1}y_{1}+\frac{8}{3}z_{1}+x_{2}y_{2}-3z_{2}+x_{3}y_{3}-3z_{3}-\\ \; & \;\;\;\;k_{3}\left(z_{1}-z_{2}-z_{3}\right). \end{array}\right.$$

The predictor-corrector method is used to numerical simulation. The initial condition is

$$\left[x_{1},y_{1},z_{1},x_{2},y_{2},z_{2},x_{3},y_{3},z_{3}\right]=\left[1,2,10,1.1,2.25,11,0.9,2.1,10.5\right],$$

$q_{r}=0.9$, and control strength $k_{1}=0.695,k_{2}=0.5,k_{3}=0.3$. The number of iterations is 3000 and the step size is 0.0055.

**Figure 14:**
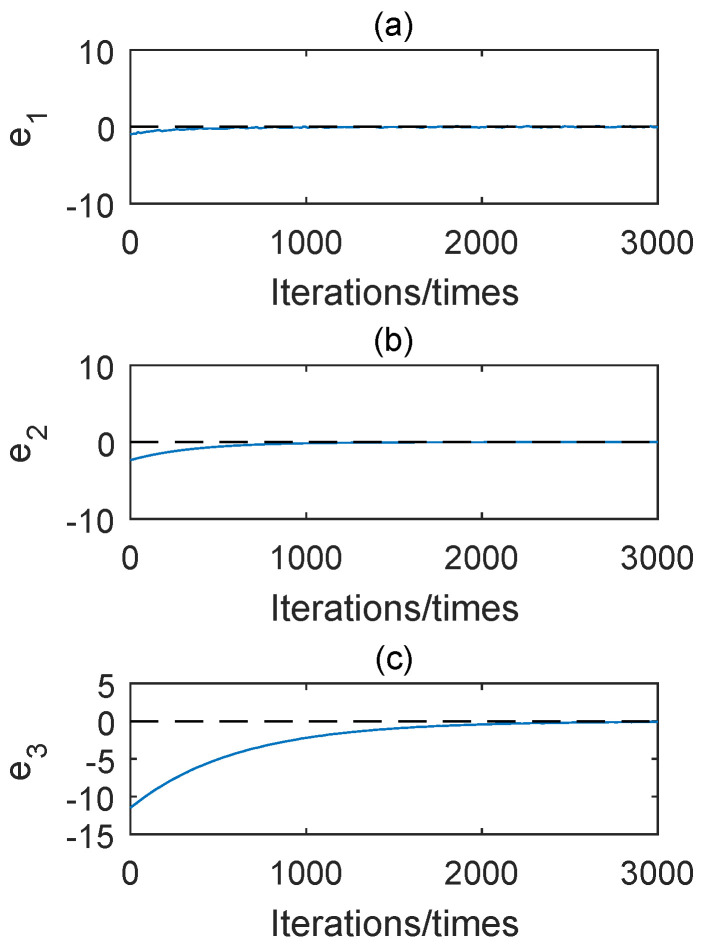
Diagram of state errors with $k_{1}=0.695,k_{2}=0.5,k_{3}=0.3$, step-size is 0.0055 and the number of iterations is 3000 and (**a**) $e_{1}=x_{1}-x_{2}-x_{3}$, (**b**) $e_{2}=y_{1}-y_{2}-y_{3}$, (**c**) $e_{3}=z_{1}-z_{2}-z_{3}$.

The diagram of state errors are shown in Figure 14. It shows the states evolutions of two drive systems and one response system. From these three pictures we can know that $e_{1}$ tends to zero in about 300th, $e_{2}=0$ in about 600th and $e_{3}$ tends to zero in about 2000th. It demonstrates that three incommensurate fractional chaotic systems quickly achieve combination synchronization with the proposed controller (25).

## 5. Conclusions

Inspired by integer order and fractional order chaotic systems, there must exist hybrid order chaotic systems. Therefore, we propose some definitions of hybrid order chaotic systems, and investigate dynamic characteristics of hybrid Lorenz, hybrid Chen, hybrid Lü, hybrid complex Lorenz systems. We find hybrid order chaotic systems have the advantages of integer order and fractional order chaotic systems, such as complicated dynamic behaviors, complex key-space and easy implementation in secure communications. By analyses of four hybrid order chaotic systems, a special relation between hybrid degree (HD), the lowest LO and the lowest TDO are discovered, which provides a simple and direct method to find the lowest fractional order chaotic system. However, the proposed conjecture is only limited in these four classic hybrid order chaotic systems and just in simulations. Generalization of this conjecture and strict mathematical proofs in other hybrid order systems deserve to be extensively study. Then, due to the special structure of hybrid order systems, incommensurate fractional combination synchronization between hybrid Chen, Lü and Lorenz systems are realized with the proposed controller by means of active control method. Numerical simulations illustrate the effectiveness and availability of the proposed control method.

In addition, there are numerous interesting things in hybrid order systems. We prove the existence of relation between hybrid degree (HD), the lowest LO and the lowest TDO in hybrid order chaotic systems. It demonstrates that the field of chaos do have some common patterns and characters. It is a completely new branch of chaos. Besides, few researchers focus on the method of searching low fractional dimension order and state variables order in chaotic systems, and the proposed conjecture can develop a directional method to seek lower order in chaotic systems. Lastly, the hybrid order complex chaotic system has more diverse dynamic behaviors, and it will increase security effect when applied to secure communication. Especially, the ergodicity and pseudorandom of hybrid order complex chaotic system can increase the diffusion of stream cipher and improve its insensitivity and avoid the defects of traditional stream cipher. Therefore, a host of works surrounding hybrid order chaotic systems are waiting to be investigated in the future. 

## Figures and Tables

**Table 1:** The **LO** and sum of different hybrid order Lorenz systems.

System	HD	LO	TDO
Integer order	3	1	3
Equation (16)	0	0.97	2.91
Equation (8)	1	0.91	2.82
Equation (9)	1	0.93	2.86
Equation (10)	1	0.95	2.9
Equation (11)	2	0.7	2.7
Equation (12)	2	0.89	2.89
Equation (13)	2	0.89	2.89

**Table 2:** Hybrid order Chen systems.

Type	Model	HD	LO	TDO
Complete	$D^{q}x=35\left(y-x\right)\;\;\;\;\;\;\;\;$			
fractional	$D^{q}y=-7x-xz+28y$	0	0.78	2.34
Chen	$D^{q}z=xy-3z\;\;\;\;\;\;\;\;\;\;$			
Hybrid	$D^{q}x=35\left(y-x\right)\;\;\;\;\;\;\;\;$			
order	$D^{q}y=-7x-xz+28y$	1	0.68	2.36
Chen	$\dot{z}=xy-3z\;\;\;\;\;\;\;$			
	$D^{q}x=35\left(y-x\right)\;\;\;\;\;\;\;\;$			
	$\;\;\;\;\dot{y}=-7x-xz+28y$	1	0.79	2.58
	$D^{q}z=xy-3z\;\;\;\;\;\;\;\;\;\;$			
	$\dot{x}=35\left(y-x\right)\;\;\;\;\;$			
	$D^{q}y=-7x-xz+28y$	1	0.995	2.99
	$D^{q}z=xy-3z\;\;\;\;\;\;\;\;\;\;$			
	$D^{q}x=35\left(y-x\right)\;\;\;\;\;\;\;\;$			
	$\;\;\;\;\dot{y}=-7x-xz+28y$	2	0.68	2.68
	$\dot{z}=xy-3z\;\;\;\;\;\;\;$			
	$\dot{x}=35\left(y-x\right)\;\;\;\;\;$			
	$D^{q}y=-7x-xz+28y$	2	0.96	2.96
	$\dot{z}=xy-3z\;\;\;\;\;\;\;$			
	$\dot{x}=35\left(y-x\right)\;\;\;\;\;$			
	$\;\;\;\;\dot{y}=-7x-xz+28y$	2	0.275	2.275
	$D^{q}z=xy-3z\;\;\;\;\;\;\;\;\;\;$			

**Table 3:** Hybrid order Lü systems.

Type	Model	HD	LO	TDO
Complete	$D^{q}x=36\left(y-x\right)\;\;\;\;$			
fractional	$D^{q}y=-xz+28.7y$	0	0.78	2.34
Lü	$D^{q}z=xy-3z\;\;\;\;\;\;$			
Hybrid	$D^{q}x=36\left(y-x\right)\;\;\;\;\;\;$			
order	$D^{q}y=-xz+28.7y\;\;$	1	0.653	2.306
Lü	$\dot{z}=xy-3z\;\;\;\;$			
	$D^{q}x=36\left(y-x\right)\;\;\;\;\;\;\;\;$			
	$\dot{y}=-xz+28.7y$	1	0.77	2.54
	$D^{q}z=xy-3z\;\;\;\;\;\;\;\;\;\;$			
	$\dot{x}=36\left(y-x\right)$			
	$D^{q}y=-xz+28.7y$	1	0.994	2.988
	$D^{q}z=xy-3z\;\;\;\;\;\;$			
	$D^{q}x=36\left(y-x\right)\;\;\;\;\;\;\;\;$			
	$\dot{y}=-xz+28.7y$	2	0.66	2.66
	$\dot{z}=xy-3z\;\;\;\;\;\;$			
	$\dot{x}=36\left(y-x\right)$			
	$D^{q}y=-xz+28.7y$	2	0.995	2.995
	$\dot{z}=xy-3z\;\;$			
	$\dot{x}=36\left(y-x\right)\;\;\;\;$			
	$\dot{y}=-xz+28.7y$	2	0.277	2.277
	$D^{q}z=xy-3z\;\;\;\;\;\;\;\;\;\;$			

**Table 4:** Hybrid order complex Lorenz systems.

Type	Model	HD	LO	TDO
Fractional	$D^{q}x=10\left(y-x\right)\;\;\;\;\;\;\;\;\;\;$			
complex	$D^{q}y=28x-y-xz\;\;\;\;\;$	0	0.959	2.877
Lorenz	$D^{q}z=\frac{1}{2}\left(\bar{x}y+x\bar{y}\right)-\frac{8}{3}z$			
Hybrid	$D^{q}x=10\left(y-x\right)\;\;\;\;\;\;\;\;\;\;$			
order	$D^{q}y=28x-y-xz\;\;\;\;\;$	1	0.908	2.816
Lorenz	$\;\;\;\;\dot{z}=\frac{1}{2}\left(\bar{x}y+x\bar{y}\right)-\frac{8}{3}z$			
	$D^{q}x=10\left(y-x\right)\;\;\;\;\;\;\;\;\;\;$			
	$\;\;\;\;\dot{y}=28x-y-xz\;\;\;\;\;$	1	0.94	2.88
	$D^{q}z=\frac{1}{2}\left(\bar{x}y+x\bar{y}\right)-\frac{8}{3}z$			
	$\;\;\;\;\dot{x}=10\left(y-x\right)\;\;\;\;\;\;\;\;\;\;$			
	$D^{q}y=28x-y-xz\;\;\;\;\;$	1	0.955	2.91
	$D^{q}z=\frac{1}{2}\left(\bar{x}y+x\bar{y}\right)-\frac{8}{3}z$			
	$D^{q}x=10\left(y-x\right)\;\;\;\;\;\;\;\;\;\;$			
	$\;\;\;\;\dot{y}=28x-y-xz\;\;\;\;\;$	2	0.531	2.531
	$\;\;\;\;\dot{z}=\frac{1}{2}\left(\bar{x}y+x\bar{y}\right)-\frac{8}{3}z$			
	$\;\;\;\;\dot{x}=10\left(y-x\right)\;\;\;\;\;\;\;\;\;\;$			
	$D^{q}y=28x-y-xz\;\;\;\;\;$	2	0.823	2.823
	$\;\;\;\;\dot{z}=\frac{1}{2}\left(\bar{x}y+x\bar{y}\right)-\frac{8}{3}z$			
	$\;\;\;\;\dot{x}=10\left(y-x\right)\;\;\;\;\;\;\;\;\;\;$			
	$\;\;\;\;\dot{y}=28x-y-xz\;\;\;\;\;$	2	0.925	2.925
	$D^{q}z=\frac{1}{2}\left(\bar{x}y+x\bar{y}\right)-\frac{8}{3}z$			
